# Dynamic ^1^H-MRS assessment of brain tumors: A novel approach for differential diagnosis of glioma

**DOI:** 10.18632/oncotarget.4899

**Published:** 2015-08-20

**Authors:** Tong Tong, Zhong Yang, John W. Chen, Jianming Zhu, Zhenwei Yao

**Affiliations:** ^1^ Department of Radiology, Fudan University Shanghai Cancer Center, Shanghai, China; ^2^ Department of Radiology, Fudan University Huashan Hospital, Shanghai, China; ^3^ Institute for Innovation in Imaging, Massachusetts General Hospital, Harvard Medical School, Boston, MA, USA; ^4^ Department of Radiation Oncology, The University of North Carolina, Chapel Hill, NC, USA

**Keywords:** clinical paper, magnetic resonance imaging, magnetic resonance spectroscopy, glioma, differential diagnosis

## Abstract

**Purpose:**

To determine whether the changes of [Cho/NAA] ratio in patients with glioma, measured by dynamic ^1^H-MRS can be used to differentiate between high-grade and low-grade gliomas.

**Materials and Methods:**

This prospective study was approved by the institutional ethics committee. Written informed consent was obtained. Forty-nine patients with biopsy-proven glioma and 20 normal control subjects were recruited in this study. The maximum [Cho/NAA] ratios, acquired at 0 min, and at 6 min, were calculated and assessed from volume of interests (VOI) in the tumor areas and in the surrounding normal tissue for each patient. Absolute difference in the [Cho/NAA] ratios, from MRS acquired at 0 and 6 min, in high-grade glioma, low-grade glioma, and control subjects were compared.

**Results:**

The maximum [Cho/NAA] ratio acquired from the tumor area at the 0 min is 6.08 ± 2.02, which was significantly different (*p* = .017) from that acquired after 6 min, 4.87 ± 2.13. The [Cho/NAA] ratio from the surrounding normal tissue area did not change significantly from spectra acquired at different times (0 min, 6 min). Absolute difference in [Cho/NAA] ratios acquired at 0 and 6 min time points were significantly higher (*P* < 0.001) in high-grade glioma (= 3.86 ± 3.31) than in low-grade glioma (= 0.81 ± 0.90), and control subjects (0.061 ± 0.026, *P* = 0.000), while there was no significantly difference in low-grade glioma and control subjects.

**Conclusions:**

Dynamic ^1^H-MRS can be useful for differential diagnosis between high-grade and low-grade gliomas as well as insight into the heterogeneity within the tumor.

## INTRODUCTION

*In vivo* magnetic resonance spectroscopy (MRS) of the brain not only provides important information on the biochemical composition and metabolism of the tissue, but can also be used to determine tissue pathology noninvasively [1]. MRS can be helpful in the evaluation of brain neoplasms. Not only can MRS show the abnormal findings in nearly 100% of brain tumors, but it is also helpful in differentiating brain tumors and characterizing metabolic changes associated with tumor progression, degree of malignancy, and response to treatment [2]. More recently, it has been shown that ^1^H-MR spectroscopy may also be useful in stereotactic biopsies and radiation therapy planning [3, 4].

However, conventional MRS has some limitations in evaluating brain tumors. Absolute quantitative thresholds in the metabolites or ratios in metabolites such as choline (Cho) and creatine (Cr) have not been firmly established to allow confident characterization of tumor grading or progression, and these numbers vary largely among different studies [5, 6, 7]. It is generally accepted that MRS offers additional information and value in diagnosing most tumors, but until recently it had not been proven that MRS information alone is sufficient to distinguish between grades of tumors of glial origin [8]. Furthermore, previous studies have shown that when using a long echo-time (TE) spectroscopy, the choline (Cho) peak area and line shape are influenced by the presence of paramagnetic contrast agents [9, 10]. These two studies reported a mean decrease of Cho peak area of 12% and 15% while no significant changes in the linewidth and chemical shift of Cho, creatine (Cr), or N-acetyl aspartate (NAA) in control brain tissue when using point-resolved spectroscopic imaging pulse sequence (PRESS, TR/TE: 1500/135ms). Gadolinium (Gd) administration thus further complicates MRS results and interpretation if the study is performed after the Gd administration.

In this study, we aim to design and test a new MRS methodology that would overcome some of the limitations with conventional MRS. We performed repeated ^1^H-MRS studies immediately one after another without any contrast agent administration. We aimed to determine whether dynamic changes in the [Cho/NAA] ratio observed from dynamic multivoxel ^1^H-MRS can be used to better differentiate between high-grade and low-grade gliomas.

## RESULTS

### Clinical and pathological findings

Pathological analysis classified 25 patients with WHO grade II glioma which were assigned to the low-grade glioma group, 16 patients with WHO grade III glioma and 8 patients with WHO grade IV glioma which were attributed to the high-grade glioma group. No WHO grade I glioma was found in our study. The details of patients and control subjects were shown in Table 1.

**Table 1:** Patient demographics

	No of patients	Age (yrs)	Sex (M/F)
Low-grade glioma	25	47.0 ± 12.5	14/11
High-grade glioma	24	58.4 ± 10.5	13/11
Control subjects	20	50.0 ± 12.5	10/10

### Dynamic ^1^H-MRS findings in phantom studies

**Figure 1:**
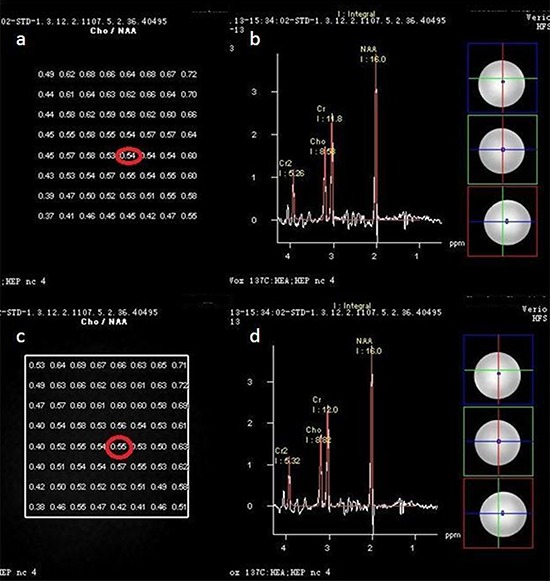
In phantom study totally 64 points to points Cho/NAA ratio in 0 min's scan a. did not differ from 6 min's 1c The Cho/NAA ratio in one point in 0 min's scan 0.54, b. did not differ from the relevant point in 6 min's scan (0.55, 1 **d.**)

In phantom studies a total of 64 points were selected, and the point to point analysis of [Cho/NAA] ratio on the 0 min scan (0.56 ± 0.08) did not differ significantly from that of the 6 min scan (0.55 ± 0.08, *p* = 0.526), as shown with results in Table 2, Figure 1.

**Table 2:** Comparison of each point value of Cho/NAA in phantom in 0 and 6 min

	*N*	Cho/NAA (0 min)	Cho/NAA (6 min)	*P*
Phantom	64	0.56 ± 0.08	0.55 ± 0.08	0.526

### Dynamic ^1^H-MRS findings in patients and control subjects

**Figure 2:**
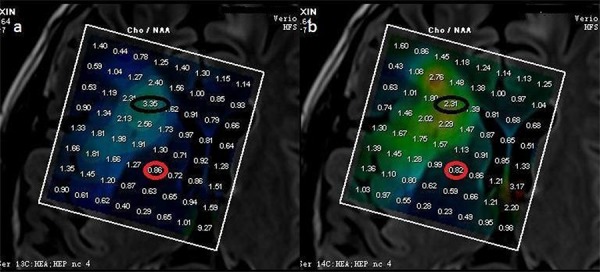
Cho/NAA of a low grade glioma (WHO II) The max Cho/NAA ratio in tumoral area in 0′min scan (**a.** 3.35) was different from the relevant value in 6 min's scan (**b.** 2.31), while the max Cho/NAA ratio in surrounding normal tissue area in 0′min scan (a, 0.86) did not differ from the relevant value in 6 min’ s scan (b, 0.82).

In patients with glioma, regardless of tumor grade, the maximum [Cho/NAA] ratio in tumor area on the 0 min scan (6.08 ± 2.02) was significantly different from that on the 6 min scan (4.87 ± 2.13, *p* =.017), while the Cho/NAA ratio in surrounding normal tissue area did not change significantly between the 0 min (0.82 ± 0.13) and 6 min scans (0.81 ± 0.14, *p* = 0.121; Table 3, Figure 2).

**Table 3:** Comparison of values for the Cho/NAA in the tumor and surrounding normal areas and in 0 and 6 min

	*N*	Cho/NAA (0 min)	Cho/NAA (6 min)	*P*
Tumor area	49	6.08 ± 2.02	4.87 ± 2.13	0.017*
Normal area	49	0.82 ± 0.13	0.81 ± 0.14	0.121

* statistically significant

### Dynamic ^1^H-MRS findings among low-grade, high-grade glioma and control subjects

**Figure 3:**
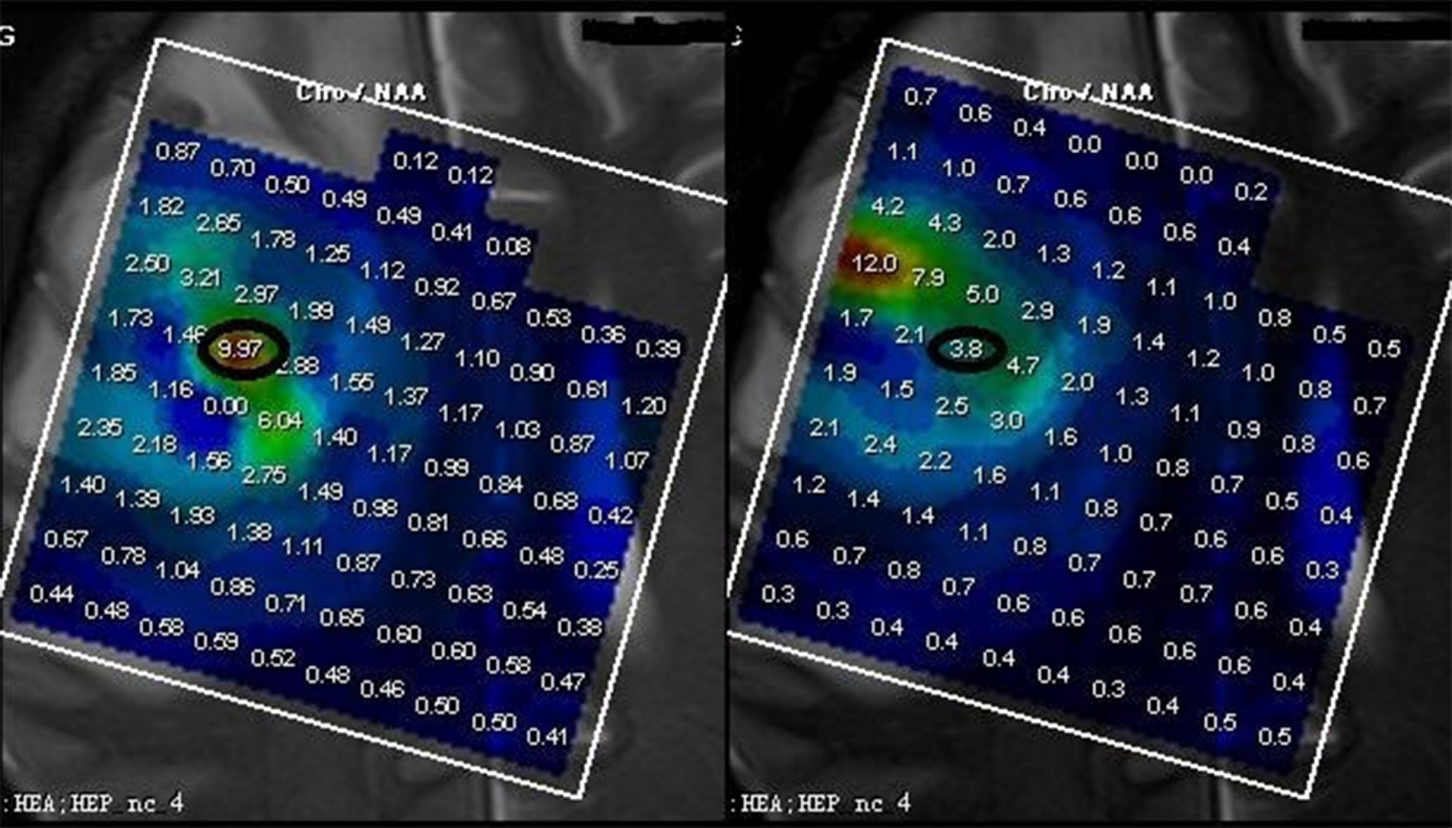
ΔCho/NAA value between 0 and 6 min was higher in high-grade glioma (6.17, Figure 4, WHO IV) than in low-grade glioma (0.24, Figure 5, WHO II) and control subjects (0.01, Figure 6)

**Figure 4:**
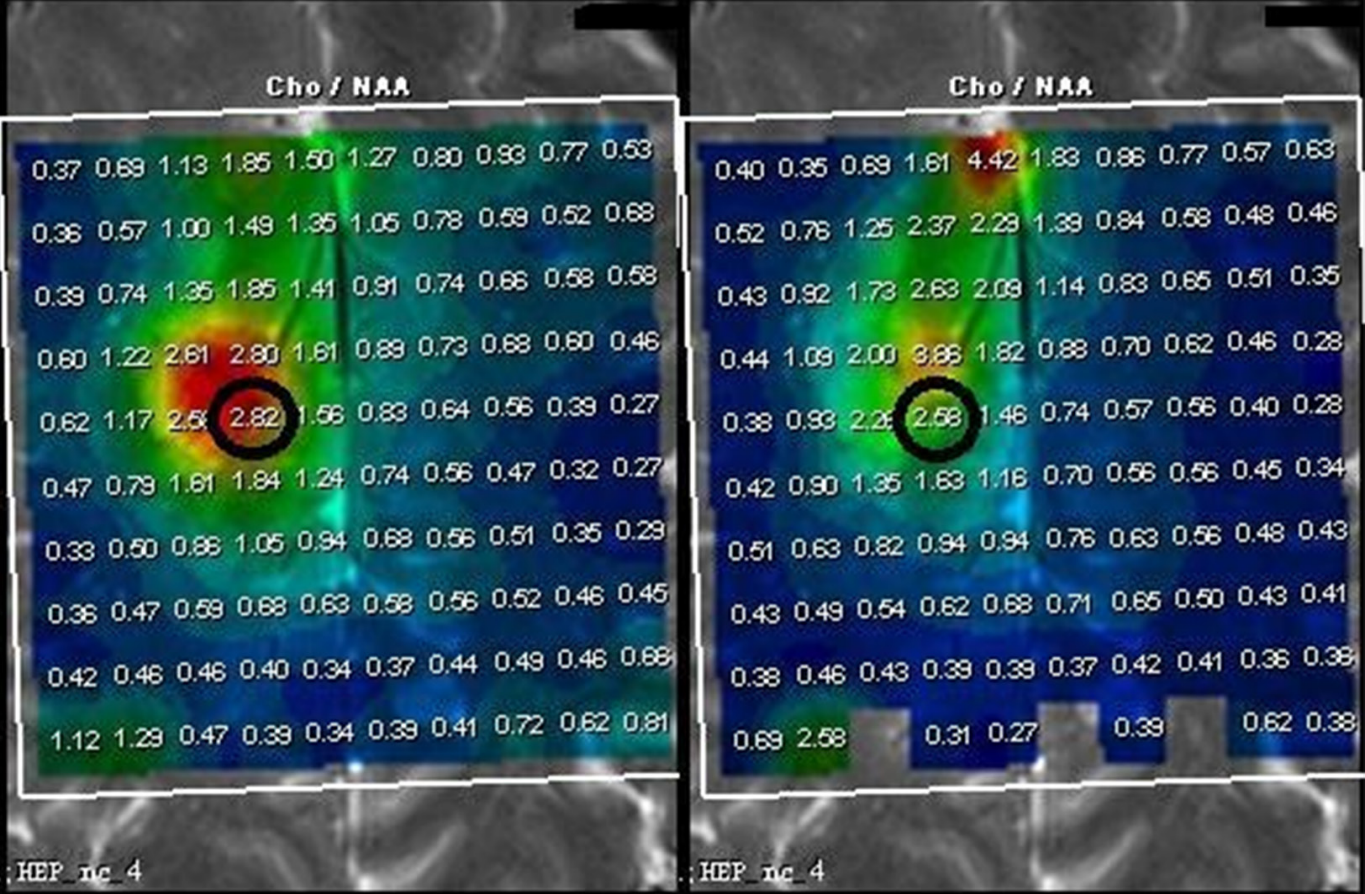
ΔCho/NAA value between 0 and 6 min was higher in high-grade glioma (6.17, Figure 4, WHO IV) than in low-grade glioma (0.24, Figure 5, WHO II) and control subjects (0.01, Figure 6)

**Figure 5:**
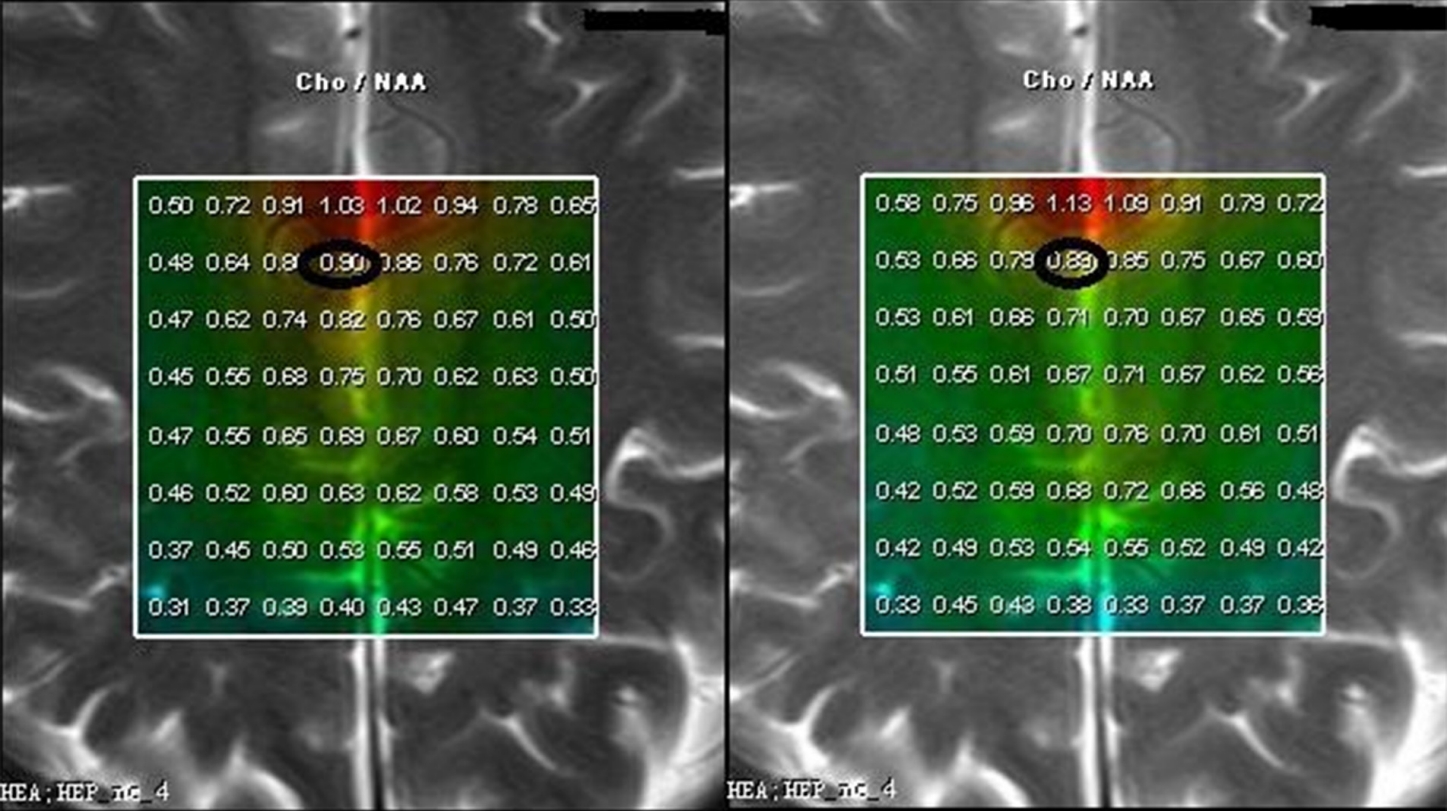
ΔCho/NAA value between 0 and 6 min was higher in high-grade glioma (6.17, Figure 4, WHO IV) than in low-grade glioma (0.24, Figure 5, WHO II) and control subjects (0.01, Figure 6)

**Figure 6:**
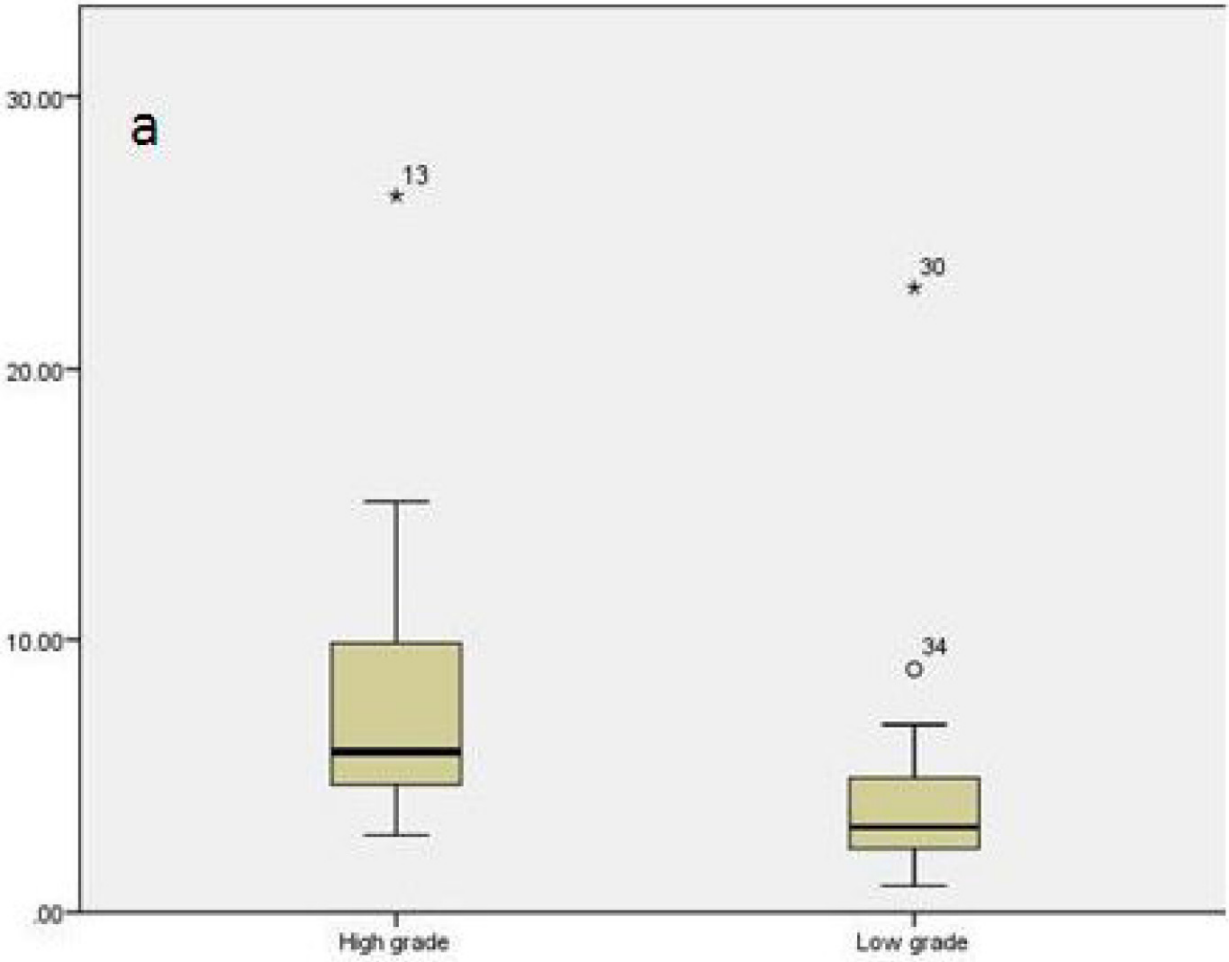
Percentile rank map of ΔCho/NAA between high-grade and low-grade gliomaΔ Cho/NAA ratiowas significantly higher in high-grade gliomas than in low-grade gliomas and here was little overlap in the two glioma groups.

**Figure 7:**
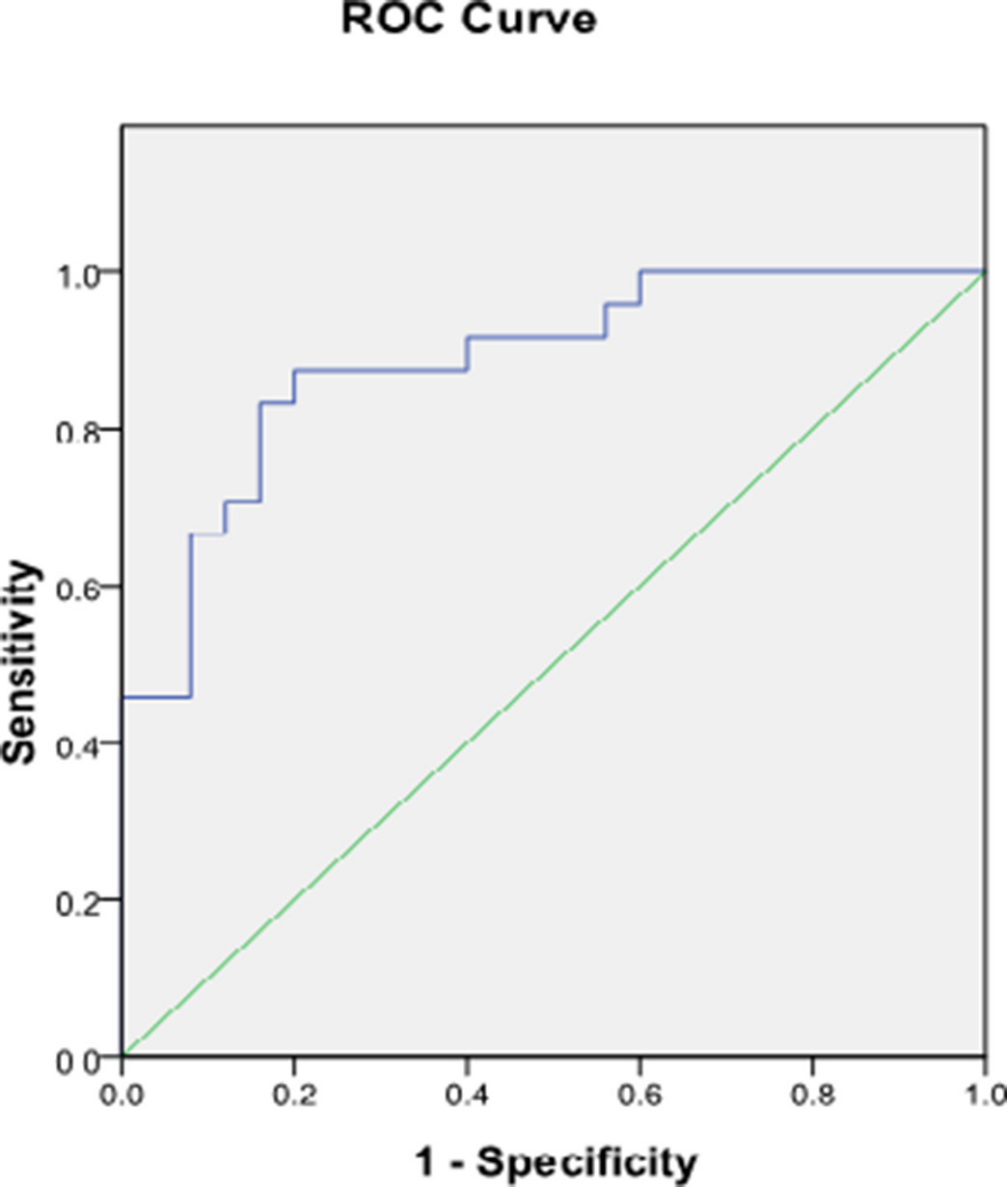
Receiver operating characteristic curve analysis for ΔCho/NAA of high-grade and low-grade glioma Area under curve shows good accuracy (0.885) for discriminating high-grade from low-grade glioma using ΔCho/NAA measurement on dynamic ^1^H-MRS maps.

Absolute difference of [Cho/NAA] ratios between 0 and 6 min were significantly higher in high-grade gliomas (3.86 ± 3.31) than in low-grade gliomas (0.81 ± 0.90, *P* < 0.001) and control subjects (0.061 ± 0.026, *P* < 0.001), while the absolute difference of [Cho/NAA] ratios between 0 and 6 min did not differ significantly in low-grade glioma (0.81 ± 0.30) and control subjects (0.061 ± 0.026,*P* = 0.224; Table 4, Figures 3, 4, 5, 6). ROC curve analysis for Δ[Cho/NAA] revealed a good value for the area under the curve (0.885) and for Δ[Cho/NAA] > 1.07, the sensitivity and specificity of the high-grade glioma were 87.5% and 80% respectively (see Figure 7)

**Table 4:** Comparison of the absolute difference of Cho/NAA values between 0 and 6 min among low-grade, high-grade glioma and control subjects

	*N*	0 min	6 min	ΔCho/NAA (0, 6 min)	*P*
High-grade glioma	24	7.79 ± 5.21	5.89 ± 5.87	3.86 ± 3.31	<0.001*
Low-grade glioma	25	4.41 ± 4.30	3.90 ± 4.20	0.81 ± 0.90	
High-grade glioma	24	7.79 ± 5.21	5.89 ± 5.87	3.86 ± 3.31	<0.001*
Control subjects	20	0.82 ± 0.13	0.81 ± 0.14	0.061 ± 0.026	
Low-grade glioma	25	4.41 ± 4.30	3.90 ± 4.20	0.81 ± 0.90	0.224
Control subjects	20	0.82 ± 0.13	0.81 ± 0.14	0.061 ± 0.026	

* indicates statistically significant

## DISCUSSION

In this study, we investigated the reproducibility of ^1^H-MRS in patients with glioma using dynamic ^1^H-MRS. Our study was focused on finding the changes of [Cho/NAA] ratios on dynamic ^1^H-MRS before applying contrast-enhanced MR, and found that, the maximum Cho/NAA ratio in the solid tumor areas on the 0 min scan was significantly different from that on the 6 min scan. On the other hand, there was no significant difference in the ratios in the surrounding normal brain tissue. We also found that Δ[Cho/NAA] ratios were significantly higher in high-grade gliomas as compared to low-grade gliomas and in control subjects. Δ[Cho/NAA] values also did not differ significantly in the low-grade glioma and control subjects.

Preoperative grading of brain tumors is necessary for better treatment planning and management. Conventional imaging findings are unreliable in the grading of brain tumors [12]. MRS studies have attempted to characterize the different histologic types and predict the degree of malignancy of brain tumors [12]. Fountas et al. [13] found that the higher the grade of the astrocytomas, the higher the Cho peak and the lower the Cr peak. They were able to differentiate between astrocytoma grades II, III and IV by using the [Cho/Cr] ratio, and proposed considering this ratio as a malignant index for the histologic grading of these tumors. We picked the voxel of the highest Cho/NAA ratio in each patient and to study the dynamic changes in this particular voxel, as some authors suggested that this voxel could represent the area of highest metabolic activity and could be used to classify the degree of the tumor malignance, and to guide stereotactic biopsy and radiation therapy [4]. Our finding of the changes in the highest Cho/NAA ratio suggest that stereotactic brain biopsy may not be best guided by MRS. Meng et al. [7] demonstrated a threshold value of 1.6 for Cho/NAA provided minimum error and 74.2%, 62.5%, 85.6%, and 44.6% for the sensitivity, specificity, PPV, and NPV for determination of a high-grade glioma. In our study threshold values of 1.07 for ΔCho/NAA was demonstrated for determining a high-grade glioma with 87.5% and 80% for sensitivity and specificity. Despite these encouraging results, there remains substantial overlap between grades, mainly due to tumor heterogeneity, a particular feature of anaplastic astrocytomas. ^1^H-MRS has also been shown to be of no advantage in the grading of tumors [14]. From our study ΔCho/NAA also showed little overlap in the two glioma groups.

The following factors may contribute to this large variation. First, Patient motion may occur at any time, and is more likely the longer the examination time. We limited subject movement by supporting the head with appropriate padding and by ensuring subject comfort and limiting the total scan time to about 12 min for the two MRS scans. Second, using a PRESS sequence is likely to be affected more by an inhomogeneous magnetic field. However, we verified the homogeneity of our magnetic field in our phantom study as well as by examining normal tissue in patients showed there was no significant difference in Cho/NAA ratio over time without the presence of a glioma. Third, postprocessing steps, especially, curve fitting, could also contribute to variations of measured concentration. In our study, fitting algorithm executed in the frequency domain fits every signal peak to Lorrentz or Gaussian curve. When the shape of signal peak was distorted because of field inhomogeneity caused by patient movement or hardware instability, fitting algorithm might not work well any more. As a result, the calculated integral area of the signal peak may not reflect the real concentration. Fourth, magnetic susceptibility effect serves on the region with high-grade tumor because of its complex inner structure. This makes shimming on these regions more difficult and the field homogeneity is easier degraded by patient's movement. As a consequence, spectral SNR will be decreased because of increased linewidth and the signal is more likely alternated by the noise. Fifth, non-homogeneity of tumor inner structure will make MRS signal more sensitive to the scanner instability, e.g. frequency drift. Even a small change on main frequency will influence the accuracy of localization, consequently alter the composition of the excited VOI and as a result the measured concentrations. Finally, dynamic ^1^H-MRS showed that, at high grade glioma, the [NAA] reduced greatly to almost to the noise level and the noise will have relatively more effect on the small NAA peak, therefore, make the [Cho/NAA] change greatly from 0 to 6 min.

Several potential limitations of our study merit comment. First, our study was performed on 3.0T MRI and high field MRI may more sensitive to head movement and micro-environment change. In the future, more studies should be carried out in 1.5T MRI to investigate whether the change exists on low field MRI scan. Second, we only selected the maximum Cho/NAA value and investigated its temporal change. We did not study the changes of the other metabolites or other metabolite ratios such as NAA/Cr and Cho/Cr. These are being carried out in the future. Finally, the small number of patients could be another limitation. A larger sample size would increase the accuracy of our findings.

## MATERIALS AND METHODS

### Patients and control subjects

MRI and quantitative multiple-voxel ^1^H-MRS were performed on consecutive patients, using the following inclusion criteria: clinical and neurologic symptoms of brain tumor or evidence of glioma on prior CT or on plain MRI, and the lesion's location would be suitable for multi-voxel ^1^H-MRS examination without generating obvious artifact. Patients with surgical clips or other metallic implants, subtentorial tumors, tumors near skull base or ventricle or tumors with hemorrhage were excluded, as were patients undergoing local brachytherapy. All patients had undergone operation and had histologically proven glioma and its grade. Forty-nine patients with glioma and 20 control subjects were recruited to this study from Jan 2013 to Dec 2013. Patients were divided into two groups, low grade and high grade, according to their pathology grading results. Control subjects were recruited from patients diagnosed with no brain tumors from clinical and MR examinations who had also no treatment. This study protocol was approved by our institutional review board, and was performed in accordance with our institutional policy on human studies and protection. All the patients enrolled in this study were given informed consent.

### Data acquisition

All patients underwent MR imaging and multi-voxel ^1^H-MR spectroscopy(MRS) on a Siemens Verio 3.0T (Siemens Medical Solutions, Germany) using a standard eight channel head coil. MR imaging included an axial 3D T1-weighted spin-echo sequence (TR/TE: 19/2.93 ms), an axial 3D T2-weighted fast spin-echo sequence (TR/TE: 3200/334 ms), and an axial 2D FLAIR sequence (TR/TE:9000/99 ms). Multi-voxel ^1^H-MR spectroscopy was performed at the site of the tumor using a PRESS chemical shift imaging (CSI) sequence (TR/TE:1700/135 ms,vector size :512, acquisition bandwidth: 1200 Hz) The transmitter frequency of RF pulse is centered around NAA. The positioning of the CSI slice was based on the location of the tumor lesions. Outer volume suppression was executed by positioning 6 saturation bands around the VOI (Volume of Interest) to suppress the signal from the outside of the VOI. An automatic 3D localized shimming technique was used to maximize the spectral resolution and homogeneity over the volume of interest. VOI shimming was then further improved by manual adjusting of the shimming parameters from shim gradients to achieve a magnitude peak width of water at half-maximum of 14 Hz or less. The VOI was smaller than the field of view at least by a factor of 1/4, to prevent aliasing artifacts. The first MRS was performed immediately after the shimming is optimized. After the first one is completed, the second set of MRS was acquired immediately after the first one. The time interval between the start of the first and the second MRS acquisition was approximately 6 min. The multi-voxel chemical shift imaging sequence produced a 10×10 transversely-oriented matrix that were defined by phase encoding scheme with a field of view (FOV) of 12 × 12 cm, resulting in an individual voxel size of 12 × 12 × 15 mm. Water resonance suppression was achieved applying chemical shift selective suppression pulses. Surrounding normal tissue was also included as a reference for postprocessing of metabolites and region definition. In control subjects, volume of interest (VOI) was selected in the centrum semiovale and also performed with the same study protocol. To ensure accurate duplication of the prescribed MRS voxels, patients and control subjects were immobilized during the MRS examination. The MR images acquired 0 and 6 min were co-registered to check that the patient positioning had not been changed.

### Phantom studies

A phantom containing a 12.5 mmol concentration of NAA, 10.0 mmol of Cr, 3.0 mmol of Cho and 5 mmol of lactate was prepared in phosphate-buffered saline (pH = 7.4). The same standard receive-only head coil was used in this study using a PRESS technique identical to that used in the patients. The measurement was performed twice (0 and 6 min) and the results were averaged to confirm the stability and reproducibility of quantitative ^1^H-MRS.

### Quantification of metabolite ratios and statistical methods

^1^H-MRS data were processed off-line with the Syngo software (Siemens Medical Solutions, Germany) to select the individual spectra of interest. The default postprocessing procedure was as followings: Before fast fourier transformation (FFT), in time domain: Residual water signal was subtracted and frequency correction was executed; hanning filter with the width of 400 ms was applied; vector size was extended to 1024 from the original size of 512. After FFT, in frequency domain: baseline correction with polynomial order of 6;automatic phase correction; curve fitting using Gaussian peak type for 3 three metabolite: NAA, Creatine and Choline. The voxels of interest selected were located on different solid tumor locations, avoiding necrotic areas, and in normal-appearing brain. For each patient, the maximum Cho/NAA ratios at 0 min and 6 min were calculated in tumor areas and in the surrounding normal tissue of each patient. We also calculated maximum Cho/NAA values in 0 min and relevant value in 6 min in control subjects. Absolute difference of Cho/NAA values between 0 and 6 min in high-grade glioma, low-grade glioma and control subjects were also calculated. The values at the edge of the VOI were excluded because of their inaccuracy.

Results of Cho/NAA values in tumor areas and the surrounding normal tissue for each patient, each point value of Cho/NAA in phantom at 0 and 6 min were compared using the paired *t*-test. Differences in absolute difference of Cho/NAA values among low-grade, high-grade glioma and control subjects were tested with ANOVA and post hoc Scheffé's tests. In all tests, *P* < 0.05 was noted as significant. Receiver operating characteristic (ROC) curves were constructed and the optimum cutoff values with the best combination of sensitivity and specificity were calculated. The overall accuracy of the diagnosis was expressed by the area under the curve, and this ranged from 0.5 to 1. A value of one implied perfect sensitivity and specificity, whereas *a* value of 0.5 implied that the model's accuracy was no better than chance. Generally, *a* value >0.7 could be interpreted as reasonable and *a* value >0.8 indicated good accuracy. Statistical analysis was performed using SPSS 16.0.

## CONCLUSIONS

Dynamic MRS is able to better distinguish between high and low grade gliomas compared to conventional MRS at 3T. Our findings may be helpful for the diagnosis and differential diagnosis of glioma as well as insight into the heterogeneity within the tumor.
